# Women-only drug treatment services and needs in Iran: the first review of current literature

**DOI:** 10.1186/s40199-016-0141-1

**Published:** 2016-02-18

**Authors:** Zahra Alam-mehrjerdi, Reza Daneshmand, Mercedeh Samiei, Roya Samadi, Mohammad Abdollahi, Kate Dolan

**Affiliations:** Program of International Research and Training, National Drug and Alcohol Research Centre, Faculty of Public Health and Community Medicine,, University of New South Wales, Sydney, Australia; Substance Abuse and Dependence Research Center, University of Social Welfare and Rehabilitation Sciences, Tehran, Iran; Department of Psychiatry, School of Behavior Sciences, University of Social Welfare and Rehabilitation Sciences, Tehran, Iran; Psychiatry and Behavioral Sciences Research Center, Department of Psychiatry, Mashhad University of Medical Sciences, Mashhad, Iran; Department of Toxicology and Pharmacology, Faculty of Pharmacy and Pharmaceutical Sciences Research Center, Tehran University of Medical Sciences, Tehran, Iran

**Keywords:** Women, Iran, Drug, Methadone, Persian Gulf

## Abstract

**Background:**

Iran (Persia) has a women-only drug treatment system. However, literature is not documented. The current study aimed to review the development of women**-**only drug treatment and harm reduction services (WODTHRS) and the factors associated with treatment entry and outcomes in Iran. The review was based on a comprehensive search for all literature focusing on WODTHRS in Iran.

**Methods:**

Data were collected by conducting systematic searching of scientific English and Persian databases and grey literature. This was done in line with Cochrane Guideline for conducting systematic reviews. Overall, 19,929 studies were found. But, only 19 original studies were included after excluding non-relevant studies.

**Results:**

The review findings indicate how WODTHRS have been developed in the past 15 years. The review findings underscore the roles of numerous factors in treatment entry such as the side effects of illicit drug use. In addition, cognitive-behavioral interventions, methadone treatment and some factors outside drug treatment such as family support increase positive treatment outcomes among women.

In contrast, financial problems as well as other factors such as insufficient medical, psychiatric and social work services hamper treatment entry and positive treatment outcomes.

**Conclusions:**

The review results highlight that eliminating barriers to treatment entry and positive treatment outcomes should be addressed. Conducting randomized controlled trials is needed to evaluate the effectiveness of WODTHRS. This issue should address the factors influencing service utilization to incorporate the best practice for women. The evaluation of the long-term efficacy of WODTHRS is a critical research gap which should be addressed in future studies.

## Background

### The origins of illicit drug use

Smoking opium has a long history in Iran which dates back hundreds of years before the tribal Arab invasion to Iran. At the time of Zoroaster, the Persian prophet, the use of some plants with euphoric effects was the main part of religious ceremonies among the Persians.

Persian physicians such as Zakariya**-**al**-**Razi and Avicenna were among the first scientists who used opium for surgery. Iran has remained a transit and consumer country for opiates because of Afghanistan, the main opium producer through the centuries [1].

### The current prevalence of illicit drug use

The total number of regular and recreational substance users is estimated to be between four and seven million [1]. It is estimated that 1,200,000-2,000,000 people are dependent on illicit drugs mainly inexpensive Afghan opium, heroin and/or methamphetamine [1, 2]. The main route of drug use is smoking [1, 2]. Other main types of illicit drugs include opium residues, hashish, tramadol and prescription opioids [1, 3, 4].

### Illicit drug use among women

According to the Ministry of Health and Medical Education (MoHME), almost ten percent of drug-dependents are women [5]. MoHME (2014) reported that there was one drug**-**using woman per eight drug**-**using men in the country. According to the report, illicit drug use is a health concern among some women [5].

In general, women are opiate smokers or poly-smokers of opiates and methamphetamine. But drug injection among women is rare because of stigma [6–10]. In addition, women with illicit drug use problem have poorer education and employment than men [11]. Women initiate illicit drug use later than men or are raised in poor environments with drug use problem [12].

## Study objectives

The provision of a drug response has been addressed for women in Iran [11, 13–15]. But, there is no previous systematic review of how women**-**only drug treatment and harm reduction services (WODTHRS) have been developed. Furthermore, the motivations and barriers associated with facilitating or hampering treatment entry and positive treatment outcomes among this group have not been documented. The current review aimed to address this gap in literature.

## Methods

### Searching procedure

The review procedure was prepared in compliance with Cochrane Guideline for conducting systematic reviews [16]. Data regarding the evidence of WODTHRS and the associated motivations and barriers were collected through a systematic literature search.

To be included in the review, years 1980–2015 were selected for searching because of a paucity of studies of illicit drug use before 1980. Studies were included if they emphasized drug treatment and/or harm reduction services for women only and their motivations and barriers for treatment. The term “motivations” refers to reasons that women report specialized for using drug treatment and/or harms reduction services. The term “barriers” refers to reasons that women do not report specialized for using drug treatment and/or harm reduction services. Studies were excluded if they were not women-only research studies.

Medical Subject Headings (MeSH) of ‘women**-**only drug services’ and ‘women**-**only harm reduction services’ were employed. MeSH subtitle headings were ‘Iran’, ‘development’, ‘treatment entry’, and ‘treatment outcomes’. Keywords added to the search parameters were ‘motivations’, ‘barriers’, ‘utilization’, and ‘access’. Searches in Google Scholar employed the phrases ‘drug treatment in Iran’, ‘harm reduction in Iran’, ‘women-only drug treatment services in Iran’, and ‘access to drug treatment and harm reduction for women in Iran’.

Based on the Guideline, English publications were retrieved through searching Web of Sciences, Medline, EMBASE, PubMed citation indexes, CINHAL, Scopus and Google Scholar. In addition, scientific Persian databases including Scientific Information Database, Magiran, Iran Medex and the website of the conference papers of Iran were searched.

Based on the Guideline, part of the searching included grey literature. This included the regional reports of the United Nations Office on Drugs and Crime, the conference abstract books of Harm Reduction Association, the National Institute on Drug Abuse and the College on Problems of Drug Dependence. The reports of MoHME and the Persian Welfare Organization were also searched.

### Search findings

**Fig. 1:**
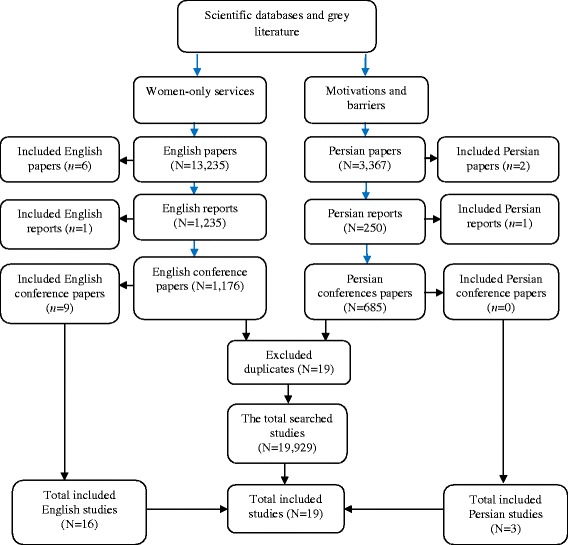
The flowchart of systematic searching

Systematic searching resulted in finding 19,929 English and Persian articles, reports and conference papers. Overall, 19 relevant studies were included. Most of the studies were related to English papers indexed in Pub Med or grey literature. Seven studies were related to the development of WODTHRS. Overall, 12 studies were related to treatment motivations and barriers. Duplicates such as editorials were excluded from the final searching (See Fig. 1).

## Results

### The development of drug treatment services

Gender-mixed drug treatment clinics were established for the first time between 1974 and 1977 that provided methadone treatment. Over the same time, 30,000 heroin and opium**-**dependents were on methadone program. But, because of Islamic views, illicit drug use was considered as a criminal activity between 1979 and 2000. Therefore, methadone treatment was not provided [1, 17, 18].

The western health policy of drug treatment was re-approved by the government in 2000 [1]. This was the result of considering illicit drug use as a health concern and the collaboration of the medical sector with the government. According to a recent MoHME report, more than 500,000 clients have received medication-assisted treatment (MAT) programs at 3,373 drug treatment centers [13].

### The development of women-only services

According to a recent report from the Welfare Organization, almost ten percent of people seeking treatment at drug treatment centers are women [19]. Women’ needs for drug treatment motivated some health policy makers to approve women-only drug services in the community [19].

The presence of some at-risk women in the community was also a motivation to approve WODTHRS. Stigma was another motivation to develop such services. A survey at eight main methadone clinics in Tehran found that only four percent of the clients were women [20]. Some women reported unwillingness to seek professional help at methadone treatment centers because of social stigma [20].

The idea of developing WODTHRS was initiated in Shiraz city near the Persian Gulf in 2001. This issue was supported by the Welfare Organization and the government [5]. The first women**-**only residential center was established by Rebirth Society (NGO) in the same city in 2002. The treatment program included 12-step meetings and a faith-based intervention. Over the same time period, “Chitgar” therapeutic community center was established by Rebirth Society in Tehran to admit women from all-over Iran [1].

In addition, “Khaneye khorshid” drop in center was established in Tehran in 2006. The center provides methadone maintenance treatment and free harm reduction services for women [1]. The first women-only methadone clinic (i.e., Persepolis clinic) was established in Tehran in 2007 [8]. Two studies at the same clinic found that only 20 % of the women reported lifetime drug treatment [9] and they needed methadone treatment [10].

Over the same time, some women especially in low socio-economic areas reported opiate dependence. This issue necessitated more opiate treatment. Therefore, some women**-**only therapeutic community and MAT programs were developed throughout the country after 2007 [11–13, 21],

Illicit drug use also led to engagements with high risk behaviors among some women which necessitated a specific response. Therefore, harm reduction services have been provided for at-risk women at women**-**only centers since 2007. Some of these centers include Atabak, Parniyan, Nader, Navid**-**e**-**Hamrazi**-**e**-**Iranians and Mikhak clinics in Tehran and other cities. The services have been based on the simultaneous provision of drug treatment and harm reduction programs [5].

The success of these centers in admitting women and the provision of WODTHRS encouraged some health policy makers to develop similar centers. Therefore, a center for at**-**risk women was established in Shiraz and Esfahan near the Persian Gulf of Iran in 2007. A study found that most of the women at the center were smokers of opiates. Women needed regular visits by infectious diseases specialists and methadone treatment. Women needed other treatment services such as an increase in the duration of receiving women-only services [21].

Furthermore, the government attempted to provide more free services for at-risk women. Therefore, five women-only harm reduction centers were established in five provinces between 2007 and 2008. Specific groups of women such as female injecting drug users were admitted at the centers [14] (See Table 1).

**Table 1:** Provided services at the centers

Clients	Harm reduction		STI^a^ management		Counseling		Referral for VCT^b^	
	n	%	n	%	n	%	n	%
Spouses of IDUs^c^	50.9	21.7	21	4.2	52	2.4	21	4.4
Spouses of prisoners	51	2.2	57	11.5	154	7.2	66	13.7
Spouses with high risk sex	10	0.4	19	3.8	394	15.8	333	6.8
Spouses of NIDUs^d^	52	2.2	55	11.1	149	7.0	55	11.4
IDUs	1366	58.1	51	10.3	130	6.1	61	12.7
NIDUs	201	8.5	142	28.6	245	11.5	120	24.9
Clients with high risk sex	134	5.7	123	24.8	948	44.6	83	17.2
Clients with recent imprisonment problem	28	1.2	28	5.6	54	2.5	43	8.9

Reference: Fahimfar et al. (2013) [14]

^a^Sexually transmitted infections

^b^Voluntary counseling and testing

^c^Injecting drug users

^d^Non-injecting drug users

**Fig. 2:**
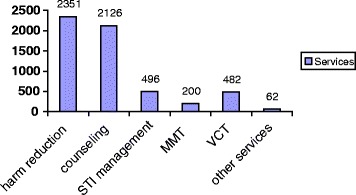
Provided services at the centers. Reference: Fahimfar et al. (2013) [14]. STI: Sexually transmitted infections

A survey found that 442 women were admitted at the centers by March 2008. Overall, 27.1 % of them reported high risk sexual behaviors. Overall, 11.3 % of them were injecting drug users. Methadone maintenance treatment, harm reduction programs such as HIV education and condom promotion were the most provided services. Overall, more than 5,000 drug treatment and harm reduction services were provided for the clients [14] (See Fig. 2).

There were 29 registered women**-**only centers in the main cities by the end of April 2014. More than 6,000 women were voluntarily admitted at the centers between 2007 and 2014. A national survey found that 2,100 women were admitted at least once at the centers to receive free WODTHRS by the end of April 2014 [14, 15].

Approximately, 45 % of women were maintained on methadone program. Counselling sessions were provided for all women. In addition, outreach teams distributed 22,000 condoms and 7,500 syringes among 3,500 at-risk women in the community. Overall, 1,762 syringes, 38,000 male condoms and 2,500 female condoms were distributed among women and their partners [14] (See Table 2).

**Table 2:** Studies related to the development of women-only services, drug treatment entry and outcomes

Study citation	Year	Sample	Study site	Main drugs	Study outcomes
Ministry of Health and Medical Education. 2014 [5]	2007**–**2014	All women (i.e., 10 % of illicit drug users)	Main cities	Any type of illicit drug especially opiates	Women-only residential centers and drop in centers such as Chitgar center and Khaneye khorshid were established for female drug users and at**-**risk women such as female sex workers and female injecting drug users.
Dolan et al. 2011a [8]	2007	78	The first women-only methadone clinic, Tehran	Opiates and poly use of opiates and methamphetamine	The first methadone clinic was established for women.
Dolan et al. 2011b [9]	2007**–**2008	78	The first women-only methadone clinic, Tehran	Opiates and poly use of opiates and methamphetamine	Only 20 % of women reported lifetime drug treatment. Women reported poor social functioning, depression, poor general health and stigma.
Dolan et al. 2012 [10]	2007**–**2008	78	The first women-only methadone clinic, Tehran	Opiates and poly use of opiates and methamphetamine	Women who had attended the clinic between 2007 and 2008 were followed in 2009–2010. Of the 78 women recruited, 40 women were followed seven months later. There was a significant reduction in heroin use at follow-up. Women needed continued methadone treatment.
Radfar. 2013 [21]	2007	15	The first two centers for health promotion among at**-**risk women, Shiraz and Esfahan, The Persian Gulf region	Opiates and poly use of opiates and methamphetamine	Women needed women-only medical, psychiatric, social and psychological services for drug treatment.
Fahimfar et al. 2013 -[14]	2007**–**2008	442	The first five harm reduction centers, five provinces	Opiates and poly use of opiates and methamphetamine	Women received drug treatment and harm reduction services at the centers.
Fahimfar et al. 2014 [15]	2007**–**2014	6,000	Five provinces	Opiates and poly use of opiates and methamphetamine	Women received free drug treatment and harm reduction services such as methadone, sterile syringes and condoms.
					Treatment motivations
Babakhanian et al. 2013 [7]	2010	69	Sixteen methadone clinics, Tehran	Opiates	Receiving information from informant sources in the community such as mass**-**media, treatment success of relatives and friends, the encouragement of healthy family members, the need for keeping family and children, an individual need to take methadone to relieve the side effects of opiate use and poor satisfaction with other drug treatments such as therapeutic community program increased treatment entry.
Ahmadan**-**Panah et al. 2014 [22]	2012	59	Ten drug treatment centers, Hamadan, western Iran	Opiates, illicit methadone and hashish	Drug withdrawal, depression, anxiety, familial problems and headaches increased treatment entry.
Alam**-**mehrjerdi et al. 2013b [23]	2008**–**2009	62	Ten methadone clinics, Tehran	Opiates	Adequate methadone dose to substitute with opiate use, counseling sessions, group therapy, individual psychological sessions, family therapy and drug education on methadone program increased positive treatment outcomes such as treatment retention, relapse prevention and the improvement of general health.
Ghasemi**-**Arganeh et al. 2014 [24]	2012	32	A women-only therapeutic community center, Isfahan	Opiates	Group motivational interviewing and life skills training increased positive treatment outcomes including the reduction of drug relapse, anxiety, depression and increased mental health.
Daneshmand et al. 2014a [25]	2011**–**2012	500	Chitgar women-only therapeutic community center, Tehran	Methamphetamine	Family support, employment, counseling and psychological services, having an ongoing program for daily activities, learning motivations to change, strategies to cope with craving, dealing with lapse, refusal skills and relapse prevention increased treatment outcomes including the improvement of general health and the provision of drug-free urine specimens.
Tafaoli**-**Masooleh. 2010 [26]	2009	70	Chitgar women-only therapeutic community center, Tehran	Methamphetamine	Cognitive**-**behavioral therapy (CBT) increased treatment outcomes including treatment retention and the provision of drug-free urine specimens in treatment.
Dehghani**-**Firooz**-**Abadi et al. 2013 [27]	2012	30	Ayandenh**-** Roshan women-only therapeutic community center, Esfahan	Opiates	CBT increased positive treatment outcomes including the provision of drug-free urine specimens and the improvement of general health.
Hadadi et al. 2014 [28]	2013	43	Four drug treatment centers and clinics, Tehran	Methamphetamine	The Matrix Model of Intensive Outpatient Treatment (CBT) increased positive treatment outcomes including treatment retention, the provision of drug-free urine specimens and the improvement of general health and psychiatric comorbidities (i.e., depression and anxiety).
					Treatment barriers
Ebrahimi et al. 2014 [29]	2012	409	Eight districts, Esfahan, central Iran	Opium	Poor treatment motivations, insufficient information and misconceptions about drug use treatment were strong barriers to treatment entry.
Shaditalab et al. 2014 [30]	2011	48	Khaneye Khorshid women drop in center, Chitgar and Congress 60 ^1^ centers, Tehran	Poly use of opiates and methamphetamine	Unemployment, low income, unstable accommodation and poor vocational training, poor physical and psychological health and poor education were barriers to achieving positive treatment outcomes. Service providers emphasized the necessity of providing social and financial supports and health insurance for increasing positive treatment outcomes such as relapse prevention.
Rahimi**-**Movaghar et al. 2011 [31]	2011	62	Chitgar center and Khaneye khorshid center, Tehran	Opiates and poly use of opiates and methamphetamine	Social stigma, poor family acceptance and low economic status were barriers to achieving treatment retention. Insufficient numbers of female medical doctors and a paucity of health counseling and educational services, living in drug**-**using environments and inadequate medical and social work services were barriers to achieving positive treatment outcomes such as treatment retention, relapse prevention and reduced psychiatric comorbidities.
Daneshmand et al. 2014b [32]	2010**–**2011	150	A central women-only drop in center, Tehran	Poly use of opiates and methamphetamine	Long duration of poly use of opiates and methamphetamine, poor family support, poor motivations to change methamphetamine use, poor participation in psychological and counseling sessions, depression, an inability to cope with everyday life pressures and inadequate skills to cope with methamphetamine craving and relapse were barriers to achieving positive treatment outcomes such as treatment compliance and the improved general health.

^1^Congress 60: a chain non-governmental organization that officially provides drug treatment and harm reduction services

### Motivation for treatment entry and positive treatment outcomes

Drug treatment entry and positive treatment outcomes may be increased by specific motivations among women. In contrast, low rates of drug treatment entry and positive treatment outcomes may emphasize specific barriers among women. Recent women-only studies in Iran found that the encouragement of others, an individual need to take methadone and poor satisfaction with some drug treatments were strong motivations for treatment entry [7].

A study found that the side effects of drug use, anxiety, depression and familial problems [22] were strong motivations for treatment entry. Two studies found that adequate methadone dose, as well as psychological services, drug education [23], motivational interviewing and life skills training [24] facilitated positive treatment outcomes. One study found that positive treatment outcomes were increased by having family support, employment and a program for daily activities [25]. Furthermore, three studies found that cognitive-behavioral interventions increased positive treatment outcomes [26–28] (See Table 2).

### Barriers to treatment entry and positive treatment outcomes

Women with poor treatment motivations or misinformation about drug treatment experience barriers to treatment entry and positive outcomes during treatment [29]. Only one study found that poor treatment motivation and lack of sufficient information about drug use were barriers to treatment entry [29]. Three studies found that financial problems as well as other factors such as insufficient women-only services were barriers to achieving positive treatment outcomes [30–32] (See Table 2).

## Discussion

Studies in western countries show that relatively low proportions of women enter drug treatment and harm reduction centers [33, 34]. WODTHRS create a unique environment that focuses on women’s issues and provides a comfortable setting in which women can discuss sensitive issues such as sex work and drug injection. Such services support women because drug treatment and harm reduction services traditionally tend to target men’ needs [35, 36].

Studies of women and drug treatment have remained undeveloped in the region that Iran has been situated [37–40]. However, Iran has initiated research on women and drug use in recent decades [41]. It should be noted that the recent development of WODTHRS in Iran is not because of the segregation of women from men [14, 15]. WODTHRS in Iran aim to address women-only needs for drug treatment [14, 15].

Gender may not be solely the predictor of treatment entry and positive treatment outcomes. However, certain drug treatment and harm reduction services may have differential impacts on treatment entry and outcomes by gender. As the numbers of female drug users may increase in Iran [14, 15], studies attempt to understand motivations and barriers associated with treatment entry and outcomes are needed in order to provide the most effective drug treatment services.

The current review findings underscore the roles of numerous factors in treatment entry. Furthermore, the review underscores that cognitive-behavioral interventions, adequate methadone dose as well as some factors outside treatment such as employment and family support increase positive treatment outcomes. In contrast, the review findings highlight that financial problems in combination with some factors such as stigma hamper treatment entry and positive treatment outcomes. Studies in the United Sates indicate that drug treatment entry and positive treatment outcomes among women are influenced by numerous individual and social factors such as social stigma, poor motivations to change and unemployment [42–44].

The highlighted role of cognitive-behavioral interventions may be related to the necessity of learning essential skills to manage drug craving and relapse. Furthermore, studies show that drug treatment is more likely to be successful if it includes both methadone and psychological services [44]. Considering the facilitating factors related to treatment entry and positive treatment outcomes, as well as the provision of CBT should be considered by health policy makers in Iran. The professional provision of cognitive-behavioral interventions is suggested to increase positive treatment outcomes. Mass**-**media and families should encourage women for drug treatment entry and utilizing harm reduction services in the community.

In contrast, the review results indicate that individual and social factors such as poor motivations to change hamper treatment entry and positive treatment outcomes. Studies in the United States indicate that women may encounter some barriers to treatment entry and engaging with treatment such as poor education about drug treatment or poor motivation to change drug use behaviors [34, 42–44]. The elimination of the barriers to treatment entry and positive treatment outcomes should be targeted by health policy makers in Iran.

It should be noted that programs with good treatment outcomes are those programs that can keep clients in treatment for long time periods [34, 44]. In addition, longer drug treatment episodes are related to positive treatment outcomes. Funded ancillary psychiatric, medical and social work services are needed to increase positive treatment outcomes among women in Iran. In addition, such programs are needed to address special needs of at**-**risk women such as trauma, rape or poly drug use [45, 46].

## Conclusion

The current review has a main limitation. The study is only based on reviewing the development of WODTHRS with an emphasis on treatment entry and outcomes in general. Conducting further reviews with an emphasis on cultural, social and ethnic barriers to drug treatment especially among women in rural areas is primarily required.

WODTHRS in Iran have been developed to address women’ needs. But, the development of similar services for women is still required. Enriching women**-**only services with enhancements such as psychiatric and employment services may increase treatment entry and positive treatment outcomes. The evaluation of the efficacy of WODTHRS versus gender**-**mixed services is primarily suggested. The main research gap is the long-term efficacy of WODTHRS which should be addressed with conducting more studies in future. Such evaluations are needed to recruit representative samples of women with longitudinal follow**-**ups.

## Abbreviations

CBT: cognitive-behavioral therapy
IDUs: injecting drug users
MeSH: Medical Subject Headings
MoHME: Ministry of Health and Medical Education
NIDUs: non-injecting drug users
STI: sexually transmitted infections
VCT: voluntary counseling and testing
WODTHRS: women**-**only drug treatment and harm reduction services
