# Erratum to: Women-only drug treatment services and needs in Iran: the first review of current literature

**DOI:** 10.1186/s40199-016-0157-6

**Published:** 2016-07-25

**Authors:** Zahra Alam-mehrjerdi, Reza Daneshmand, Mercedeh Samiei, Roya Samadi, Mohammad Abdollahi, Kate Dolan

**Affiliations:** 1 Program of International Research and Training, National Drug and Alcohol Research Centre, Faculty of Public Health and Community Medicine, University of New South Wales, Sydney, Australia; 2 Substance Abuse and Dependence Research Center, University of Social Welfare and Rehabilitation Sciences, Tehran, Iran; 3 Department of Psychiatry, School of Behavior Sciences, University of Social Welfare and Rehabilitation Sciences, Tehran, Iran; 4 Psychiatry and Behavioral Sciences Research Center, Department of Psychiatry, Mashhad University of Medical Sciences, Mashhad, Iran; 5 Department of Toxicology and Pharmacology, Faculty of Pharmacy and Pharmaceutical Sciences Research Center, Tehran University of Medical Sciences, Tehran, Iran

## Erratum

Following publication of the original article in DARU Journal of Pharmaceutical Sciences [1], it was brought to our attention that the following sentences are incorrect:

“Smoking opium has a long history in Iran which dates back hundreds of years before the tribal Arab invasion to Iran.” The sentence should read: Smoking opium has a long history in Iran which dates back hundreds of years before the brilliant era of Zoroaster.

“Therefore, a center for at-risk women was established in Shiraz and Esfahan near the Persian Gulf of Iran in 2007.” This sentence should read: Therefore, two centers for at-risk women were established in Shiraz and Esfahan near the Persian Gulf of Iran in 2007.

“In addition, such programs are needed to address special needs of at-risk women such as trauma, rape or poly drug use [45, 46].” This sentence should read: In addition, such programs are needed to address special problems of at-risk women such as trauma, rape or poly drug use [45, 46].

Also, the header “Motivation for treatment entry and positive treatment outcome” should read: Motivations for treatment entry and positive treatment outcomes.

We apologize for the inconvenience.
